# Implementing advance care planning in heart failure: a qualitative study of primary healthcare professionals

**DOI:** 10.3399/BJGP.2020.0973

**Published:** 2021-05-05

**Authors:** Markus Schichtel, John I MacArtney, Bee Wee, Anne-Marie Boylan

**Affiliations:** Department of Public Health and Primary Care, University of Cambridge, Cambridge.; Unit of Academic Primary Care, Warwick Medical School, University of Warwick, Coventry.; Oxford Centre for Education and Research in Palliative Care, Oxford.; Nuffield Department of Primary Care Health Sciences, University of Oxford, Oxford.

**Keywords:** advance care planning, heart failure, primary health care, qualitative research, shared decision making

## Abstract

**Background:**

Advance care planning (ACP) can improve the quality of life of patients suffering from heart failure (HF). However, primary care healthcare professionals (HCPs) find ACP difficult to engage with and patient care remains suboptimal.

**Aim:**

To explore the views of primary care HCPs on how to improve their engagement with ACP in HF.

**Design and setting:**

A qualitative interview study with GPs and primary care nurses in England.

**Method:**

Semi-structured interviews were conducted with a purposive sample of 24 primary care HCPs. Data were analysed using reflexive thematic analysis.

**Results:**

Three main themes were constructed from the data: ACP as integral to holistic care in HF; potentially limiting factors to the doctor–patient relationship; and approaches to improve professional performance. Many HCPs saw the benefits of ACP as synonymous with providing holistic care and improving patients’ quality of life. However, some feared that initiating ACP could irrevocably damage their doctor–patient relationship. Their own fear of death and dying, a lack of disease-specific communication skills, and uncertainty about the right timing were significant barriers to ACP. To optimise their engagement with ACP in HF, HCPs recommended better clinician–patient dialogue through question prompts, enhanced shared decision-making approaches, synchronising ACP across medical specialties, and disease-specific training.

**Conclusion:**

GPs and primary care nurses are vital to deliver ACP for patients suffering from HF. HCPs highlighted important areas to improve their practice and the urgent need for investigations into better clinician–patient engagement with ACP.

## INTRODUCTION

Heart failure (HF) is a major cause of mortality and morbidity worldwide, with an increasing proportion of patients suffering from refractory HF requiring palliative care.^1^^,^^2^ Improving quality of life for these patients is a fundamental goal of HF management in national and international guidelines.^3^^–^^5^ Patients with HF often suffer from a high symptom burden, unpredictable disease trajectory, and severe prognosis.^2^^,^^3^ Studies show that advance care planning (ACP) can improve the quality of life of these patients,^6^^–^^9^ and is defined as a voluntary process that helps patients in sharing their personal values and goals of future care in order to safeguard their care preferences in case they become seriously ill.^10^ As such, ACP is significant in identifying early palliative care needs and preparing for the end of life.^11^ While ACP is widely advocated in HF, merely 7% of HF decedents, compared with 50% of cancer patients, had their palliative care needs recognised,^12^ and only 8%–10% of patients had the opportunity to participate in ACP, mainly because healthcare professionals (HCPs) found ACP difficult.^13^^,^^14^ As a result, the majority of HF patients receive suboptimal palliative care.^3^^,^^15^

It is widely accepted that GPs and primary care nurses are central to engage with ACP because of their pivotal role in the provision of continuous supportive and palliative care in the community.^16^^,^^17^ Moreover, patients and carers think that GPs and nurses should have a prominent role in ACP.^18^ However, what approaches and interventions GPs and primary care nurses perceive as helpful to improve their engagement with ACP in HF has not been investigated to date.

Previous research into implementing ACP in HF has primarily focused on interventions targeting patients to engage with ACP,^19^^–^^25^ but not on primary HCPs. The few studies that have examined primary care professionals’ experiences with ACP in HF demonstrate concerns about the timing, initiation, conduct, and recording of ACP conversations, worries about competency of disease-specific communication skills, and the lack of resources.^26^^–^^28^ To increase HCPs’ engagement with ACP in HF, the field needs to identify and develop effective ways that improve clinical practice.^29^^,^^30^ This study explores and reports on the views and ideas of GPs and community nurses on how to improve their engagement with ACP when working with patients suffering from HF in England.

The specific objectives of this aspect of the study reported were: to explore how GPs and community nurses working with patients suffering from HF understand ACP and their role within ACP; and to identify factors that may facilitate or impair primary HCPs’ engagement with ACP in palliative care for HF.

**Table:** How this fits in

Research has shown that primary care healthcare professionals (HCPs) find it difficult to engage with advance care planning (ACP) in heart failure (HF), affecting patient care. This study found qualitative evidence drawn from HCPs’ practical experience providing insights into their work challenges of engaging with ACP in HF. These findings address the recognised evidence gap in the literature, of which approaches perceived as helpful by HCPs to improve their engagement with ACP in HF include: a patient-led question prompt list, a shared decision-making tool, an ACP prompt between GPs and cardiologists, and disease-specific, practice-based ACP training.

## METHOD

The authors undertook an interpretive and descriptive study using semi-structured interviews with primary HCPs in the South of England, including rural and urban communities. Semi-structured interviews were seen as a suitable approach to generate personal in-depth findings based on HCPs’ experience in sensitive topics such as end-of-life care.^31^ This study explored the experiences of GPs and primary care nurses on how to improve their engagement with ACP in HF. The interview series involved HCPs who delivered clinical services and ACP, consisting of full- and part-time GPs, district, practice, and end-of-life care nurses, as well as HF specialist nurses. This study focused on primary care staff because evidence suggests that primary HCPs are considered central professionals in the management of continuous and end-of-life care.^32^ The study was approved by the first author’s institutional ethics committee. Written informed consent was given by all study participants. Research participants and their corresponding data were pseudonymised.

### Recruitment

Likely participants were identified from publicly accessible information on GP practice websites in the South of England, the local Clinical Research Network, by word of mouth, and through professional contacts. Purposive sampling aimed for a maximum variation in participants,^33^ seeking a range of HCPs (for example, full- and part-time GPs, salaried, academic, and out-of-hours GPs, community HF specialist nurses, practice and district nurses, a variety of settings [urban and country practices], a degree of experience [for example, HCPs with little or no experience in ACP, or HCPs looking after patients suffering from HF], and age, sex, and ethnicity). The authors approached 29 potential participants with a letter of invitation, the participant information sheet (see Supplementary Appendix S1), a reply, and a consent form (see Supplementary Appendix S2) requesting information concerning demographics, their clinical role, and numbers of years in clinical practice (Table 1). Participant recruitment would be stopped after reaching data saturation, a point where no new themes emerged, with a high rate of duplication or recurrence of responses.^34^

**Table 1.** Characteristics of participants (*N* = 24)

**Composition of sample**	***n***	**%**
**GPs**	17	70.8
Full and part time	9	37.5
Salaried	5	20.8
Locum	2	8.3
Out of hours	1	4.2

**Nurses**	7	29.2
District and practice nurse	5	20.8
Heart failure specialist nurse	2	8.3

**Sex**		
Female	15	62.5
Male	9	37.5

**Ethnicity**		
White British	23	95.8
Asian British	1	4.2

**Age range, years**	29–68	—

### Data collection and coding

Semi-structured, in-depth interviews were conducted between April and September 2016 by one researcher, who is a GP and clinical academic with experience in qualitative research. One-off audiorecorded interviews were undertaken at a place of the participant’s choice, lasting between 35 and 60 mins. An interview topic guide (see Supplementary Appendix S3) explored participants’ experience with ACP in HF, perceived barriers to engaging with ACP, their management of ACP in recent cases, and suggestions on what might help them to improve their engagement with ACP. The interview guide was piloted and refined during the first three interviews of the study. Initially, multiple questions about their experience with ACP, and barriers and facilitators to its implementation were developed. Pilot testing was performed by qualitatively testing the questions using a form of cognitive interviewing. The purpose of cognitive interviewing and testing the topic guide was to investigate how well questions perform when asked of participants. This allowed the authors to select the optimal question for each topic and refine the wording to produce a field-test version.^35^ The pilot topic guide required only minor adaptations, and qualitative data during the pilot interviews were included in the final data analysis.

All interviews were conducted face-to-face at the participants’ place of work. No new themes were identified after 21 interviews, with a high rate of recurrence of topics and no new development of codes or themes. However, three further interviews were undertaken to ensure data saturation. All interviews were audiorecorded, then independently transcribed verbatim by an external professional transcription service soon after recording. All transcripts were checked for accuracy by the interviewer. A sample of transcripts was independently checked for accuracy and coded by a sociologist and non-clinician with significant expertise in thematic analysis to enhance the credibility of data collection and analysis.

### Data analysis

Interview data were interpreted inductively using Braun and Clarke’s six phases of thematic analysis:^36^ data familiarisation; generating initial codes; constructing themes; reviewing potential themes; defining and naming themes; and producing the report. Transcripts were entered into NVivo (version 11), coded, and thematically analysed. A clinical academic led the analysis. He kept a reflexive diary, reflecting on the influence of his own professional background on data analysis, including deviant case analysis.

To increase rigour, three members of the research team with backgrounds in sociology, nursing, and extensive experience of end-of-life research had ongoing involvement with and input into the data analysis.^37^^,^^38^ Two of these researchers compared and reflected on the early and subsequent coding decisions together with the interviewer. A third researcher reviewed findings and provided new insights throughout the coding process. The authors regularly attempted to identify deviant cases by actively seeking out interview responses that did not conform to the views of the majority of interviewees. These iterative steps informed the interviewer’s interpretive analysis.

## RESULTS

**Figure 1.**
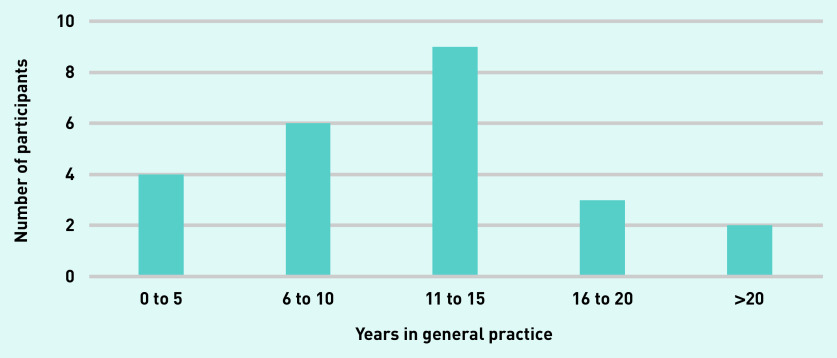
**Years in general practice.**

Twenty-five participants out of 29 invitees replied to express an interest in taking part. A total of 24 HCPs participated in the interviews. Data saturation was ensured after 24 interviews, making the participation of the remaining participant redundant. The majority of interviewees were GPs, females, and had a white British origin (Table 1). They displayed a varying level of experience in general practice (Figure 1). Nearly one-third of interviewees were nurses, including practice, HF specialist, and district nurses.

**Figure 2.**
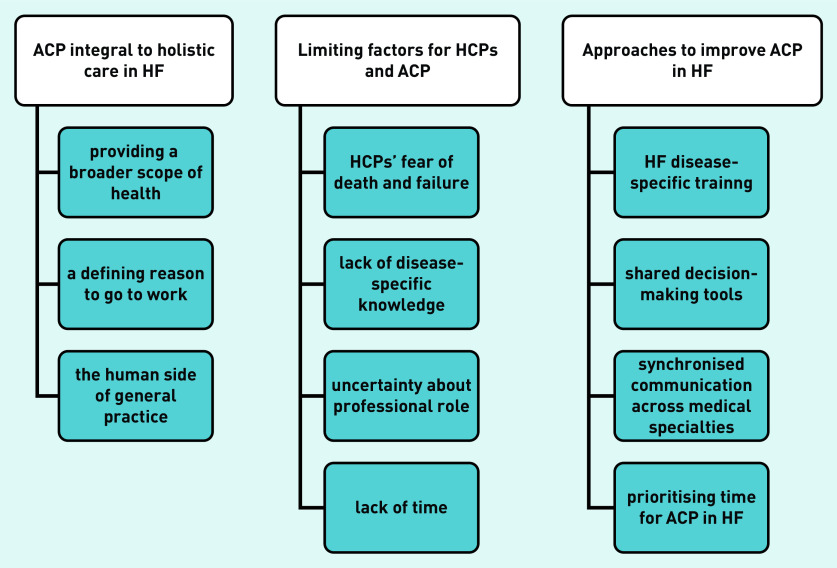
*Interrelated themes.* *ACP = advance care planning. HCP = healthcare professional. HF = heart failure.*

The authors agreed on three interrelated main themes and their subthemes (Figure 2): ACP as integral to holistic care in HF; potentially limiting factors to the doctor–patient relationship; and approaches to improve HCPs’ ACP performance.

### ACP as integral to holistic care in HF

#### Providing a broader scope of health

Many HCPs saw end-of-life care in HF as synonymous with providing holistic care, which, in itself, represented a strong benefit and motivation to do their job. One GP said:

*‘I think for GPs on the whole, the thing that floats our boat, is the idea of holistic care. It is the idea of not just firefighting the moment, but actually thinking about a broader definition of health.’*

(Responder [R]21)

#### A defining reason to go to work

Being able to provide holistic care was not only a strong but also a defining reason for some nurses to go to work. One district nurse explained:

*‘I think end-of-life is what every district nurse goes to work for. We do like the other little bits and pieces that we do, but if they were going to take end-of-life away from us, I think we would all be up in arms. If they took bloods away, that’s fine. Not end-of-life. For me, that’s a privilege.’*

(R24)

#### The human side of general practice

Another GP said:

*‘I think these conversations are really important to have. For me, it’s the kind of human side of general practice. This is the stuff that kind of matters. For me, it’s trying to provide good-quality care for people; care that is holistic and care that involves their families. I mean, it’s a cliché, but you only get one chance to get it right. It’s also really easy for it to go wrong … But having these conversations, I find it really satisfying.’*

(R19)

### Potentially limiting factors to the doctor–patient relationship

#### HCPs’ fear of death and failure

Many GPs and nurses indicated that their own fear of death and dying may be one of the most powerful reasons why they did not engage with ACP in HF. One nurse described her corresponding emotions:

*‘But what makes this conversation tricky is the fear of death, both the death of our patients and our own deaths, which I think varies subconsciously for most of us. That’s the biomedical approach which I think still sees death as a failure, as an enemy rather than a natural part of life.’*

(R14)

Some HCPs feared that delivering ACP could irrevocably damage their doctor–patient relationship. A GP said:

*‘My biggest fear was getting it wrong and upsetting the person and not being able to recover that and damaging the relationship and not being able to go there again. But, actually, I watched my colleague do it a few times and it went badly, and she recovered it. And there are ways that you can still have a rapport with somebody and still help them to have that conversation, even if you’ve kind of messed it up to start with. We are all human, so you don’t get it right every time.’*

(R13)

All GPs and nurses acknowledged that death and dying were a common experience as part of their professional role, but only very rarely were they comfortable with talking about death and dying with their patients. One GP said:

*‘End-of-life can be quite a taboo subject for some doctors. It’s something we are a bit frightened about. That clashes with the expectation of medicine* — *that medicine is about curing problems, about extending life … Nobody is stepping back, saying, “You are dying, and shall we plan for that so you have a good death?”’*

(R2)

For one GP, this was due to his perception that having an end-of-life conversation was equal to admitting the failure of medicine and his failure in the role as a clinician:

*‘I think it’s very difficult if your lifetime approach has been around successes and length of life. It’s almost an acknowledgement of the failure of medicine, isn’t it, to have that conversation.’*

(R10)

#### Lack of disease-specific knowledge

A number of HCPs admitted that they lacked disease-specific knowledge for ACP in end-stage HF. The unpredictable disease trajectory of HF compared with other end-of-life conditions, such as cancer, made it more difficult for a number of HCPs to know when to have an end-of-life care conversation with their patients. One GP said:

*‘With cancer, I actually find it much easier to know that I am within a few weeks or days of someone dying because I find the disease trajectory much more predictable. And therein lies the problem with HF and why my ACP experience with HF is limited because I find it much harder to predict and getting harder.’*

(R5)

One nurse said:

*‘Discussions around ACP in HF are the more difficult conversations to have. I ask myself, “Do I have the skills? Do I want to be that person that if somebody is enjoying the here and now starts talking about the end-of-life.” Obviously, these are the harder things to talk about, because people don’t necessarily want to be thinking about it.’*

(R7)

#### Uncertainty about roles or responsibilities

Some GPs perceived HF nurse specialists as much better qualified to deal with the palliative care dimension of the disease:

*‘If there is a specialist palliative care nurse or HF nurse in end-of-life, then expecting a GP to be better than the specialist nurse teams would be a surprise to me. If that is the presumption then it’s incorrect, isn’t it? I think we are definitely not any better than the specialist nurse teams.’*

(R4)

GPs also perceived initiating an ACP conversation with an HF patient amounted to undermining the role of their secondary care colleagues. One GP described a strong sense of deference towards cardiologists as a reason for not having ACP conversations with HF patients:

*‘I think the relationship could be a lot better between cardiologists and GPs. Cardiologists need more prompting to acknowledge when people have very advanced HF in conversations with the patient as well as in the letter to the GP. They should give the GP permission to have an end-of-life conversation. I think a lot of cardiologists would be surprised that that is the case, that GPs might feel they need permission, in a sense, to have that conversation.’*

(R9)

#### Lack of time

A lack of time was seen by many GPs and nurses as another important barrier to deliver ACP. One GP said:

*‘You are not going to do ACP in ten minutes. It’s going to take you two hours to do this properly. You just have to make it work. I know it’s difficult.’*

(R12)

One nurse commented:

*‘Even if the skills are there, the time generally isn’t. Responding in a timely manner or having enough time to do the complex work is very difficult.’*

(R7)

### Approaches to improve HCPs’ ACP performance in HF

#### HF disease-specific training

When asked what would help HCPs overcome these barriers, many participants expressed the need to receive training in end-of-life care for HF to increase their knowledge base on disease-specific facts. To make the importance of ACP memorable to trainees and establish their knowledge base about HF more permanently, one experienced district nurse suggested that students should be given an exercise that applies ACP to their own lives:

*‘I think, right at the beginning of everybody’s training, there needs to be stuff in there about palliative and end-of-life care and ACP, and they start with their own advance care plan.’*

(R20)

In order for training to be effective and sustainable, it needed to fit into existing educational programmes of GPs and nurses. Ideally, the training should take place at their local surgery. In this way, learning activities would also reach those professionals, who normally would not attend a palliative care event.

#### Shared decision-making tools

When asking an HF nurse, what would help her most in deciding whether to start ACP for a patient or not, she replied:

*‘I very much believe in using templates before consultations in specific areas. But the templates don’t always give you the wording to communicate well with patients. And so, that’s perhaps something that could be developed … When you are discussing a patient, a template that covers each individual patient’s palliative care needs, which can be completed during the meeting — that would be a useful tool.’*

(R3)

Another GP suggested:

*‘The easiest thing of all for me is when a patient asks us a question. And there have been instances where particularly sort of older gents were saying: “Is my time up?” That clear.’*

(R5)

A number of HCPs concurred with that view and would welcome patients taking the initiative in asking them questions to start a conversation. One nurse thought that patients using question prompt lists would help her in knowing what they wanted to talk about:

*‘If a patient came to me in clinic and handed me a list of questions and said, “This is the question I have for you today” … I would like that.’*

(R7)

One nurse emphasised:

*‘I find it helpful if prompts ask them about who they want at their bedside in their last days. Are there specific things they want to be treated for or not be treated for? They might want to be treated for a chest infection, or they don’t want to be treated for it … Be sure that it’s the patient’s document and it’s their plan, and it’s not the nurse’s plan. We are looking at making documentation more personalised across the district nursing team.’*

(R20)

Another GP said:

*‘I think that shared decision-making tools would be excellent. Not least of all to facilitate the conversation, because it is so much easier. So, that’s one of the pluses of a form. You could give it to the patient in advance and they could write quite a bit on it. And that gives them the opportunity to put as much or as little, and tick boxes about what they did or didn’t want to discuss. That really helps the clinician to know where the patient is at.’*

(R21)

#### Synchronised communication across medical specialties

Some GPs suggested that the signal for the right timing to have an ACP conversation should come from the cardiologist or the HF specialist nurse, since they were seen as the subject experts. One GP said:

*‘I would find it incredibly helpful to receive from a cardiologist or HF specialist nurse the information or prompt that they are happy for me to have an end-of-life conversation with a patient … Having the sort of permission to do that would actually be really helpful, because it would make me say, “Okay, good, they think that there is not much else that can be done. She’s on maximum medical therapy to help and this is about, you know, the focus is on managing symptoms”, and that’s just so much of an easier conversation if it feels like we are all sort of singing from the same hymn book.’*

(R8)

Possible solutions for creating such a prompt were discharge summaries, patient notes, or patient passports that highlighted the importance of having an ACP conversation. A nurse suggested:

*‘The discharge summary can have a box, which serves both as a prompt and as a means of communication between the cardiologist and the GP saying, “Has any end- of-life conversations gone on?” Wouldn’t that be good?’*

(R5)

All participants highlighted the importance of being able to have telephone or face-to-face conversations with colleagues to keep updated on patients’ end-of-life care. Yet, structural changes to clinical services meant that some nurses were no longer based in the same building as GPs, and communications were less direct.

#### Prioritising time for ACP

A common option for GPs to create more time was to have an ACP conversation in the context of a home visit:

*‘I think having two hours after surgery to go and see this family was vital to have a successful end-of-life conversation. I see these kinds of patients after work or on the way home. It was helpful to have as much time as we needed to discuss these things. We continued with the discussion until it naturally came to an end. This is incredibly valuable.’*

(R4)

Overall, there were no deviant cases among the common views or perspectives of HCPs. As a method of training HCPs to deliver ACP, one GP cautioned against using the traditional form of role-play and suggested an alternative:

*‘The thing about role-play is that it puts you on the spot when you have to do it with colleagues. I think it is quite a painful experience. I think doing it differently, doing it like watching a video of somebody doing it and then discussing what good phrases they have used is a bit less threatening in some ways.’*

(R8)

## DISCUSSION

### Summary

Twenty-four GPs and nurses provided insights into their experience and engagement with ACP in HF. Their own fear of death and dying, a lack of disease-specific communication skills, and uncertainty about the right timing were significant clinician barriers to not engaging with ACP in HF. To improve their engagement with ACP, primary HCPs suggested better clinician–patient dialogue through question prompts, enhanced shared decision-making approaches, synchronised coordination of care across medical specialties, and HF-specific training in ACP.

### Strengths and limitations

The views of 24 HCPs with diverse roles in primary care and a range of experience in ACP and HF generated detailed insight into identifying ways of improving clinical practice. The combination of rural and urban practice settings generated valuable findings that might be transferable across the UK.

The interviewer’s clinical role as a GP helped participant–researcher rapport by understanding the existing working culture.^37^ However, to ensure rigour, a second, non-clinical researcher contributed to data analysis.^38^ Two other researchers participated in the key stages of data analysis in order to obtain different perspectives and, therefore, to enhance the credibility of the analysis.^36^

A limitation of the interview sample was that it included only HCPs from primary care. Secondary care HCPs such as cardiologists were excluded from the interview study. Their experience and ideas about how to best implement ACP in HF may differ from their primary care colleagues and warrant further investigation.

### Comparison with existing literature

The literature concurs with the views of a number of the study participants that ACP can contribute to the job satisfaction of HCPs,^39^ improve the quality of life of patients, and can contribute to holistic patient care, especially if it is carried out by trained clinicians working in multidisciplinary teams.^40^

A previous systematic review on clinician barriers and facilitators to HF ACPs^27^ concluded that training HCPs in the delivery of ACP might be as important as enabling patients to start an ACP conversation. However, novel findings from this interview study identify a new level of detail on ways of effectively training HCPs in ACP for patients with HF, their perception of the value of shared decision-making tools, and how communication across medical specialties could be synchronised.

Studies indicate that ACP training can occur in several ways. In conjunction with formal education, whether by face-to-face teaching or distance learning, the use of mentorship styles of training are significant, so that junior staff could directly observe and learn from their more experienced colleagues.^41^ While most of this study’s HCPs acknowledged that undertaking ACP should be part of everyone’s responsibility, it was evident that the required skills came only as a result of regular practice. High levels of competence and confidence in delivering ACP were usually the outcome of much exposure to managing patients with terminal illnesses, a special interest in end-of-life care, or working together with like-minded colleagues.^42^ Additionally, given the time constraints of routine clinical practice, study participants recommended that ACP training needed to fit into existing educational programmes of GPs and nurses to be sustainable. Ideally such training should take place at their place of work. A Cochrane review corresponded with these findings, indicating that practice-based outreach has the potential to reach even those HCPs, who normally would not attend a training event requiring time to travel.^43^

A number of GPs and nurses identified practical ways to address their time constraints to undertake ACP even in the context of their busy clinical practice. Importantly, there was a general consensus among participants that these conversations could not be rushed and were, by nature, time intensive. Going on a home visit was a common approach to creating more time for ACP. The literature concurs with these results, while adding that sequencing home visits or appointments may have the benefit of spreading the emotional burden of such a sensitive topic.^44^

This study demonstrated a perceived hierarchy gap between GPs and cardiologists, which can pose a barrier to working across medical specialties for the implementation of ACP. A literature review about interprofessional team working has highlighted the need for shared goals to enable effective team working.^45^ GPs suggested an ACP communication prompt between them and their cardiology colleagues to enable primary–secondary care team working to ensure the right timing of initiating ACP.

HCPs confirmed the emotional impact that ACP conversations had on them.^41^ While all GPs and nurses acknowledged that death and dying were a common experience as part of their professional role, only very rarely were they comfortable with talking about death and dying with their patients. Further, the unpredictable disease trajectory of HF compared with other end-of-life conditions, such as cancer, made it more difficult for a number of HCPs to know when to have an end-of-life care conversation with their patients. Therefore, a number of HCPs suggested that patients could take the initiative in asking them questions. This may have the potential to relieve some of the HCP fears when engaging with ACP, indicating the right timing of having such a conversation.

### Implications for practice

Developing shared decision-making tools in HF or a prompt list for patients were advocated by HCPs. These tools might mitigate against HCPs’ fears of causing unnecessary alarm and provide a platform for ACP conversations to take place. Given the paucity of shared decision-making tools in HF,^46^ the need for their development seemed supported by this study. While these tools might be helpful, the literature also cautions against using them rigidly by allowing the conversation to degenerate into a tick-box exercise.^47^ ACP conversations need to remain person-centred and tailored to the individual patient.^48^

This clinician interview series provided some key suggestions from GPs and nurses on how to overcome some barriers to their engagement with ACP in HF. HCPs recommended a prompt list for patients, a shared decision-making tool, a communication prompt between primary and secondary colleagues, and practicebased ACP training as approaches to improve their current practice. Findings from this research can contribute to the design of corresponding interventions to improve the implementation of ACP in HF.
